# Single-Chain Soluble Receptor Fusion Proteins as Versatile Cytokine Inhibitors

**DOI:** 10.3389/fimmu.2020.01422

**Published:** 2020-07-13

**Authors:** Aurora Holgado, Harald Braun, Kenneth Verstraete, Domien Vanneste, Nico Callewaert, Savvas N. Savvides, Inna S. Afonina, Rudi Beyaert

**Affiliations:** ^1^Center for Inflammation Research, Unit of Molecular Signal Transduction in Inflammation, VIB, Ghent, Belgium; ^2^Department of Biomedical Molecular Biology, Ghent University, Ghent, Belgium; ^3^Center for Inflammation Research, Unit for Structural Biology, VIB, Ghent, Belgium; ^4^Department of Biochemistry and Microbiology, Ghent University, Ghent, Belgium; ^5^Center for Medical Biotechnology, VIB, Ghent, Belgium

**Keywords:** IL-33, IL-4, IL-13, allergy, inflammation, cytokine, biologics

## Abstract

Cytokines are small secreted proteins that among many functions also play key roles in the orchestration of inflammation in host defense and disease. Over the past years, a large number of biologics have been developed to target cytokines in disease, amongst which soluble receptor fusion proteins have shown some promise in pre-clinical studies. We have previously shown proof-of-concept for the therapeutic targeting of interleukin (IL)-33 in airway inflammation using a newly developed biologic, termed IL-33trap, comprising the ectodomains of the cognate receptor ST2 and the co-receptor IL-1RAcP fused into a single-chain recombinant fusion protein. Here we extend the biophysical and biological characterization of IL-33trap variants, and show that IL-33trap is a stable protein with a monomeric profile both at physiological temperatures and during liquid storage at 4°C. Reducing the N-glycan heterogeneity and complexity of IL-33trap via GlycoDelete engineering neither affects its stability nor its inhibitory activity against IL-33. We also report that IL-33trap specifically targets biologically active IL-33 splice variants. Finally, we document the generation and antagonistic activity of a single-chain IL-4/13trap, which inhibits both IL-4 and IL-13 signaling. Collectively, these results illustrate that single-chain soluble receptor fusion proteins against IL-4, IL-13, and IL-33 are novel biologics that might not only be of interest for research purposes and further interrogation of the role of their target cytokines in physiology and disease, but may also complement monoclonal antibodies for the treatment of allergic and other inflammatory diseases.

## Introduction

Cytokines are small proteins secreted by immune cells that bind to specific high affinity cell surface receptors. This subsequently initiates an intracellular signaling cascade, which culminates in the activation of transcription factors to induce expression of specific genes important for different cellular activities. Cytokines are considered key modulators in host defense against external threats or injury, as well as in initiating and regulating both innate and adaptive immunity. Dysregulated cytokine signaling leads to the development of inflammatory and autoimmune diseases, as well as cancer. Several biologics preventing the activity of cytokines have been developed as new protein-based therapeutics for inflammatory diseases. These include monoclonal antibodies neutralizing specific cytokines or blocking their receptor, recombinant decoy receptors targeting cytokines, as well as recombinant proteins that can either be cytokine receptor agonists or antagonists (1). Because of their versatility, efficacy and relative ease of large-scale manufacturing, humanized monoclonal antibodies have been the preferred treatment choice and several have emerged as blockbuster drugs. For example, the anti-TNF antibody Adalimumab reduces inflammation in a number of autoimmune diseases including Crohn's disease, ulcerative colitis, plaque psoriasis, rheumatoid arthritis and ankylosing spondylitis (2, 3); Dupilumab is an anti-IL-4Rα monoclonal antibody used to treat allergic diseases (4); and Mepolizumab blocks IL-5 and ameliorates the symptoms of patients suffering from severe eosinophilic asthma (5). On the other hand, recombinant soluble receptor-based cytokine antagonists have also found their way into the clinic. For instance, the dimeric soluble TNFR2 receptor-based immunoglobulin (Ig) G1 Fc fusion protein Etanercept is a blockbuster drug against ankylosing spondylitis, plaque psoriasis, psoriatic arthritis, and rheumatoid arthritis (6). Similarly, Rilonacept is a dimeric fusion protein consisting of the ligand-binding domains of the extracellular portions of the human IL-1 receptor component (IL-1R1) and IL-1 receptor accessory protein (IL-1RAcP) linked to an IgG1 Fc region, which is approved for clinical treatment of cryopyrin-associated periodic syndromes (7, 8). Another cytokine receptor based biologic is Aflibercept, which is a recombinant fusion protein consisting of vascular endothelial growth factor (VEGF)-binding portions from the extracellular domains of human VEGF receptors 1 and 2, that are fused to a human IgG1 Fc portion, and which is used for the treatment of age-related macular degeneration (9). Finally, Anakinra is an example of a recombinant form of the natural anti-inflammatory cytokine IL-1 receptor antagonist (IL-1Ra) that competes with IL-1 for binding to its receptor, and that was approved for the treatment of rheumatoid arthritis as well as a number of autoinflammatory diseases due to excess IL-1 (10). Despite these clinical successes, existing biologics often only help a subset of patients or suffer from other limitations such as side effects and resistance due to the development of anti-drug antibodies, indicating the need for new or complementary approaches.

IL-33, a member of the IL-1 family of cytokines, is best known for its role in the activation of T helper (Th)2 cell-mediated (also known as type 2) immunity at mucosal body surfaces, where it is released from epithelial and endothelial cells exposed to allergens and other cellular stress factors (11). IL-33 is also considered to function as an “alarmin,” activating various immune cells (T cells, macrophages, innate lymphoid cells type 2) upon binding to its cell surface receptor ST2 [reviewed in Braun et al., (11)]. The pathological role of IL-33 is most firmly established in the case of asthma, supported by a large body of experimental data ranging from transgenic overexpression or local intra-tracheal administration of recombinant IL-33, *IL-33* or *ST2* gene ablations, and pharmacological inhibition of the IL-33 signaling pathway in mice (11, 12). Consequently, IL-33-blocking agents are actively developed as new therapeutic biologics. Such agents include anti-IL-33 and anti-ST2 monoclonal antibodies as well as recombinant decoy receptors corresponding to the extracellular part of the IL-33 receptor ST2 (known as soluble ST2 or sST2). For instance, Regeneron Pharmaceuticals, in collaboration with Sanofi, entered Phase 2 clinical trials for asthma, chronic obstructive pulmonary disease and atopic dermatitis with an anti-IL-33 antibody (REGN3500). Another anti-IL-33 monoclonal antibody, Etokimab (AnaptysBio), is also under evaluation or completed Phase2a trials for moderate-to-severe adult atopic dermatitis, chronic rhinosinusitis with nasal polyps, asthma and peanut allergy (13). Moreover, two ST2-targeting monoclonal antibodies, AMG282 (Genentech) and GSK3772847 (formerly CNTO 7160; GlaxoSmithKline), are also in Phase2 clinical trials for asthma.

IL-33 binds with relatively low affinity to its cognate cell surface receptor ST2, which then serves as a binding platform to recruit the co-receptor IL-1RAcP, thus forming a heterodimeric high affinity signaling competent receptor complex (14). This principle led us to engineer a recombinant fusion protein (referred to as “IL-33trap”), comprising the extracellular domains of ST2 (sST2) and IL-1RAcP (sIL-1RAcP) interconnected by a flexible linker, which was anticipated to behave as a high affinity single molecule antagonist of IL-33 cytokine activity. Indeed, IL-33trap showed dramatically enhanced binding affinity to IL-33 when compared to recombinant sST2, which corresponds to the natural decoy receptor for IL-33. Moreover, IL-33trap efficiently prevented the development of airway inflammation and airway hyperreactivity in a murine asthma model (15). More recently, IL-33trap was also shown to suppress colorectal cancer tumor growth by decreasing infiltrating tumor-associated macrophages that negatively impact tumor immunity (16). In the present study, we focus on the further biophysical and biological characterization of the IL-33trap. We also report the generation and characterization of another single chain receptor fusion-based cytokine modulator, termed IL-4/13trap, which exhibits great capacity to inhibit IL-4 and IL-13. Altogether, our data illustrate that single-chain soluble receptor fusion proteins against IL-4, IL-13 and IL-33 are novel biologics that are not only of interest as research tools, but may also complement monoclonal antibodies for the treatment of allergic and other inflammatory diseases.

## Materials and Methods

### Expression Plasmids and Recombinant Proteins

Plasmids have been deposited at the BCCM/GeneCorner plasmid collection (www.genecorner.ugent.be) hosted by our department. p4x-STAT6-Luc2P (LMBP09396), which contains a STAT6-driven luciferase reporter gene, was purchased from Addgene. pNFconluc, which contains an NF-κB–driven luciferase reporter gene, was a gift from Dr. A. Israel (Institut Pasteur, Paris, France), and pACTbgal (LMBP4341) was from Dr. J. Inoue (Institute of Medical Sciences, Tokyo, Japan). Construction of human and mouse IL-33traps, as well as production of mouse IL-33trap in HEK 293 FreeStyle cells, were described previously (15). Full length human IL-33 was PCR amplified from a human cDNA library and ligated into pCR-Blunt II-TOPO. Splice variants were made by inverse PCR reaction. Subsequently, IL-33 full length and splice variants with a C-terminal 6xHis-tag were PCR amplified and cloned into pJExD by homologous recombination (CloneEZ). The basic bacterial expression vector pJExD, which allows crystal violet-induced expression, was made by modifying the commercial vector pET-Duet1 as follows: lacI and the first T7 promoter and lacO binding site (Eco47III—BamHI) were replaced with a synthetic sequence containing an eilR expression cassette and the crystal violet inducible JExD promoter with eilR binding sites (17). Expression of IL-33 splice variants in *Escherichia coli* BL21 was induced by addition of 100 nmol/L crystal violet (Sigma-Aldrich, Belgium) for 3.5 h at 37°C. Cells were collected by centrifugation, lysed and soluble IL-33 variants were purified using immobilized metal affinity chromatography using Ni-NTA sepharose (IBA Lifesciences, Germany). Production of truncated mouse IL-33 (residues 109–266) in *Escherichia coli* BL21 has been described previously (15). Briefly, protein expression was induced with 1 mmol/L isopropyl β-D-1-thiogalactopyranoside, followed by overnight incubation at 28°C. The bacterial pellet was harvested by means of centrifugation, resolubilized, and lysed by means of sonication. The lysate was centrifuged, and soluble IL-33 was purified from the supernatant by using immobilized metal affinity chromatography, followed by size exclusion chromatography (SEC). Protein concentration was determined with a bicinchoninic acid (BCA) protein assay kit.

The ectodomains of the human IL-13Rα1 and IL-4Rα were PCR amplified from a human cDNA library and cloned into pEF6-MycHisA to generate pEF-ShIL13Ra1 and pEF-ShIL4R, respectively. To generate the IL-4/13trap expression plasmid, a human IL-4Rα PCR fragment amplified from pEF-ShIL4R and a linker sequence of 20 times repeating Gly-Gly-Ser (GGS) triplets amplified from pCLG-Duba (LMBP6610) were cloned into the pEF-ShIL13Ra1 vector via 3-way ligation. All constructs were confirmed using DNA sequencing analysis. Human IL-4/13trap and human IL-33trap were produced in HEK293T cells and purified from the medium fraction by immobilized metal affinity chromatography using Ni-NTA sepharose (IBA Lifesciences, Germnay). To reduce the glycosylation complexity and heterogeneity, murine IL-33trap was also produced in suspension growth serum-free adapted HEK293 GlycoDelete cells (18).

### Cytokine Bioassays

HEK293T cells (gift from Dr. Hall, Department of Biochemistry, University of Birmingham, United Kingdom) were seeded at 4 × 10^4^ cells/well in 24-well plates and cultured in Dulbecco's modified Eagle medium supplemented with 10% FCS and 2 mmol/L L-glutamine. The next day, cells were transiently transfected by calcium phosphate precipitation with specific IL-33, IL-4, or IL-13 cytokine receptor expression plasmids. For the IL-33 bioassay, cells were co-transfected with the NF-κB reporter plasmid pNFconluc and the constitutively expressing β-galactosidase plasmid pACTbgal. For the IL-4 and IL-13 bioassay, cells were co-transfected with STAT6 and the STAT6 reporter plasmid p4x-STAT6-Luc2P, as well as the constitutively expressing β-galactosidase plasmid pACTbgal. 24 h later, cells were stimulated with recombinant IL-33, IL-4, or IL-13 for 5 h. For cytokine neutralization experiments, cytokines were incubated for 30 min at room temperature with specific cytokine trap inhibitors before addition to the cells. Cell lysates were analyzed for luciferase activity and normalized based on β-galactosidase levels to correct for potential differences in transfection efficiency.

### Measurement of Protein Aggregation via SEC-MALLS

Potential protein aggregation was measured via size-exclusion chromatography (SEC) coupled with multi-angle laser light scattering (MALLS). Protein samples were injected onto a Superdex 200 Increase10/300 GL column (GE Healthcare), with PBS pH 7.4 as running buffer at 0.5 ml/min, coupled to an inline ultraviolet-detector (Shimadzu), a multi-angle light scattering miniDAWN TREOS instrument (Wyatt) and an Optilab T-rEXrefractometer (Wyatt) at 25°C. A refractive index increment (dn/dc) value of 0.185 ml/g was used for protein concentration and molecular mass determination. Data were analyzed using the ASTRA6 software (Wyatt). Correction for band broadening was applied using parameters derived from BSA injected under identical running conditions. For the analysis of IL-33traps, conjugate analysis was performed using theoretical protein extinction coefficients and a dn/dc value of 0.160 ml/g for the glycan modifier.

### Measurement of Thermostability

Thermostability was measured by ThermoFluor® assay as described (19). Protein samples were diluted in a final volume of 16 μl of PBS buffer and 1 μl of 300X SYPRO Orange (Invitrogen™, Thermo Fisher Scientific) was added. Each experiment was run as a technical triplicate, with a triplicate blank measurement without test protein. Fluorescence in function of the temperature was recorded in a 348-well LightCycler® 480 (Roche Life Science) from 25 to 95°C at 0.02°C/s. Melting temperatures (T_m_) were calculated as the V50 value of a Boltzmann sigmoidal curve fitted to the averaged data points of the three replicates in each experiment. Onset temperatures (T_o_) were calculated as previously described (20). For graphing, the raw data sets were averaged, blank corrected and then normalized (minimal value at 0%, maximal value at 100%), using Prism 7 Software (GraphPad).

## Results

### Use of a Flexible Linker Allows the Generation of an Fc-Less Single-Chain IL-33trap

**Figure 1:**
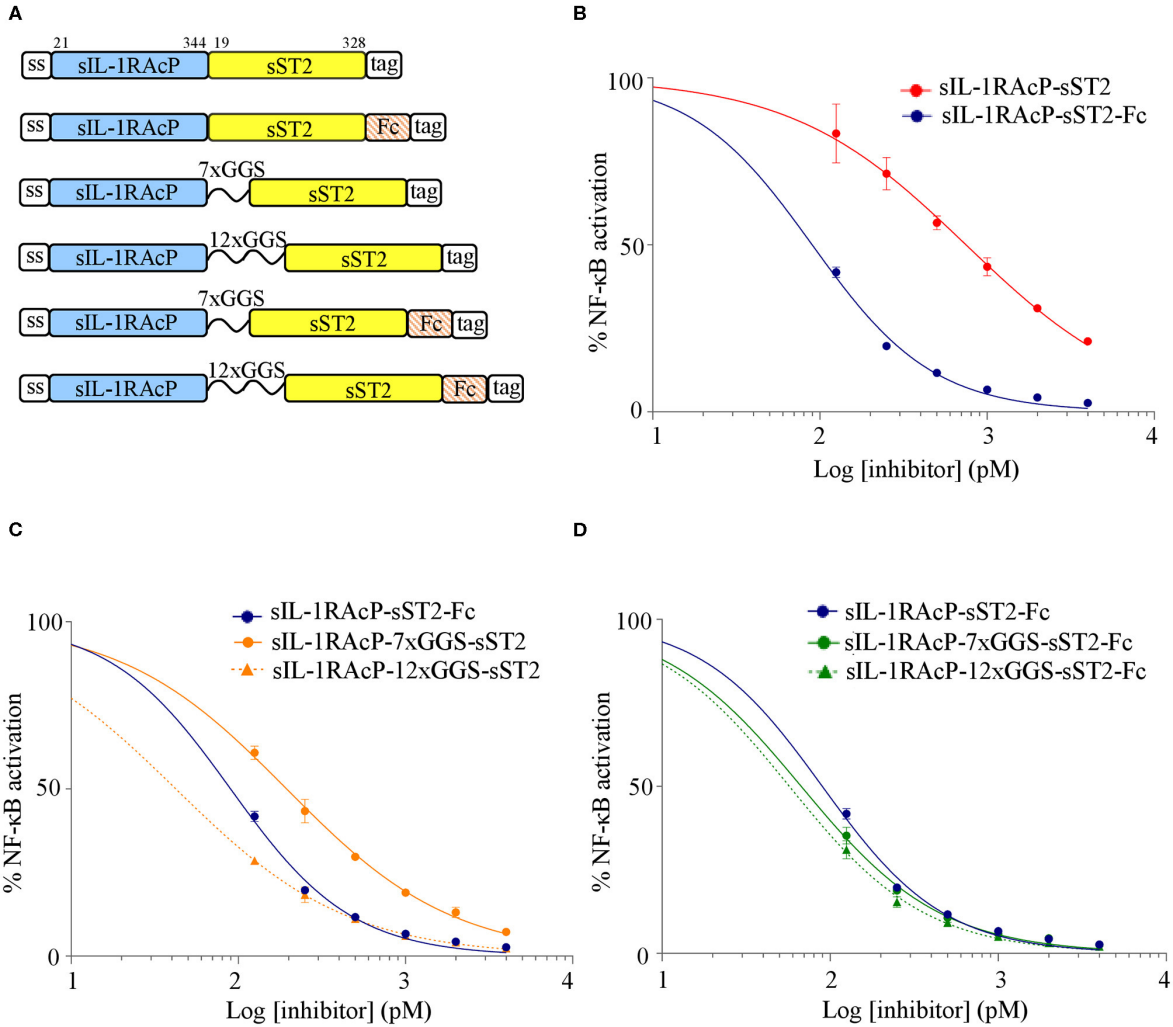
Comparison of the effect of Fc fusion and introduction of a 12xGGS linker on the inhibitory capacity of human IL-33trap. **(A)** Schematic representation of human IL-33trap constructs (numbers indicate the amino acid boundaries of the receptor ectodomains). **(B–D)** Effect of different fusion proteins on IL-33 induced NF-κB activation. HEK293T cells were treated with recombinant human IL-33 that was pre-incubated with equimolar concentrations of the indicated fusion proteins over a range of inhibitors/target ratios and assayed for NF-κB activity as described in Material and Methods. Values represent means ± SE of technical triplicates. Results are representative of at least two independent experiments.

Recombinant cytokine decoy receptor-based biologics such as Etanercept, Rilonacept, and Aflibercept all contain the Fc portion of human IgG1. Fusion to an Fc domain allows dimerisation of the two receptor subunits and provides manufacturing, biological and pharmacological advantages *in vivo*, including established large-scale affinity purification, half-life extension due to pH-dependent binding to the neonatal Fc receptor (FcRn) (21). For the construction of IL-33trap we used an alternative approach that avoids the need for an Fc to induce receptor dimerisation. More specifically, we cloned a flexible GGS linker sequence between sST2 and sIL-1RAcP to ensure the intramolecular formation of an active sST2/sIL-1RAcP heterodimeric receptor complex (15). Although at that time we did not compare the bioactivity of our linker containing IL-33trap with an IL-33trap variant that does not contain a linker, it is likely that the linker is needed for optimal domain orientation and heterodimerisation between both receptor subunits and consequently the bioactivity of IL-33trap. To further compare the effect of a linker sequence with the effect of fusion to an Fc-moiety on IL-33trap bioactivity, we generated recombinant human IL-33trap variants in which both receptor subunits (sST2 and sIL-1RAcP) were either not separated by a linker or contained a 7x or 12x GGS linker (shown empirically to enable the formation of biologically active IL-33trap), and compared these with the corresponding IL-33trap constructs that also contain a human IgG1 Fc domain at the C-terminus (Figure 1A).

The antagonistic activity of different human IL-33trap constructs was analyzed by measuring their ability to inhibit IL-33-induced activation of an NF-κB-dependent luciferase reporter gene in HEK293T cells that were made IL-33 responsive by transient transfection with human ST2. Prior to cell stimulation, recombinant human IL-33 was incubated for 30 min with equimolar concentrations of each human IL-33trap variant over a range of inhibitors/target ratios. An IL-33trap without Fc and GGS linker had an almost 10-fold reduced activity compared to a similar GGS-less construct fused to an Fc (IC_50_ of 748 pM and 90 pM, respectively; Figure 1B), suggesting that the Fc-moiety allows the optimal IL-33trap conformation for ligand binding, most likely by mediating dimerisation of two IL-33trap molecules. Importantly, inclusion of a 7x GGS or 12xGGS linker sequence instead of an Fc also enhanced the antagonistic effect of the IL-33trap (IC_50_ of 194 pM and 42 pM, respectively; Figure 1C), with sIL-1RAcP-12xGGS-sST2 being slightly more potent than sIL-1RAcP-sST2-Fc (IC_50_ of 42 vs. 90 pM; Figure 1C). Combined use of a 12xGGS linker and an Fc fusion in a single IL-33trap construct (IC_50_ of 57 pM) did not much further change the potency of a 12xGGS-only or Fc-only construct (IC_50_ of 42 or 90 pM, respectively; Figures 1C,D). However, additional Fc fusion leads to a more potent molecule in the case of a shorter 7xGGS linker (IC50 67 vs. 194 pM; Figures 1C,D). Collectively, these data illustrate that fusion of either a GGS linker sequence or an Fc-moiety increases the IL-33 antagonistic activity of an sIL-1RAcP-sST2 fusion protein by enabling, respectively, intramolecular and intermolecular interactions between sST2 and sIL-1RAcP that are necessary for the formation of a high affinity IL-33 binding complex. The lower potency of a 7xGGS containing construct (IC_50_ of 194 pM) compared to a 12xGGS (42 pM) construct suggests that a longer linker increases molecular flexibility, favoring a more optimal intramolecular interaction between sST2 and sIL-1RAcP. Importantly, our data illustrate that insertion of a flexible 12xGGS linker between both IL-33 receptor subunits allows the formation of a single-chain fusion protein with high IL-33 antagonistic activity, circumventing the need for Fc fusion and Fc-mediated dimerisation.

### IL-33trap Is Biophysically Stable During Liquid Storage and at Physiological Temperature

**Figure 2:**
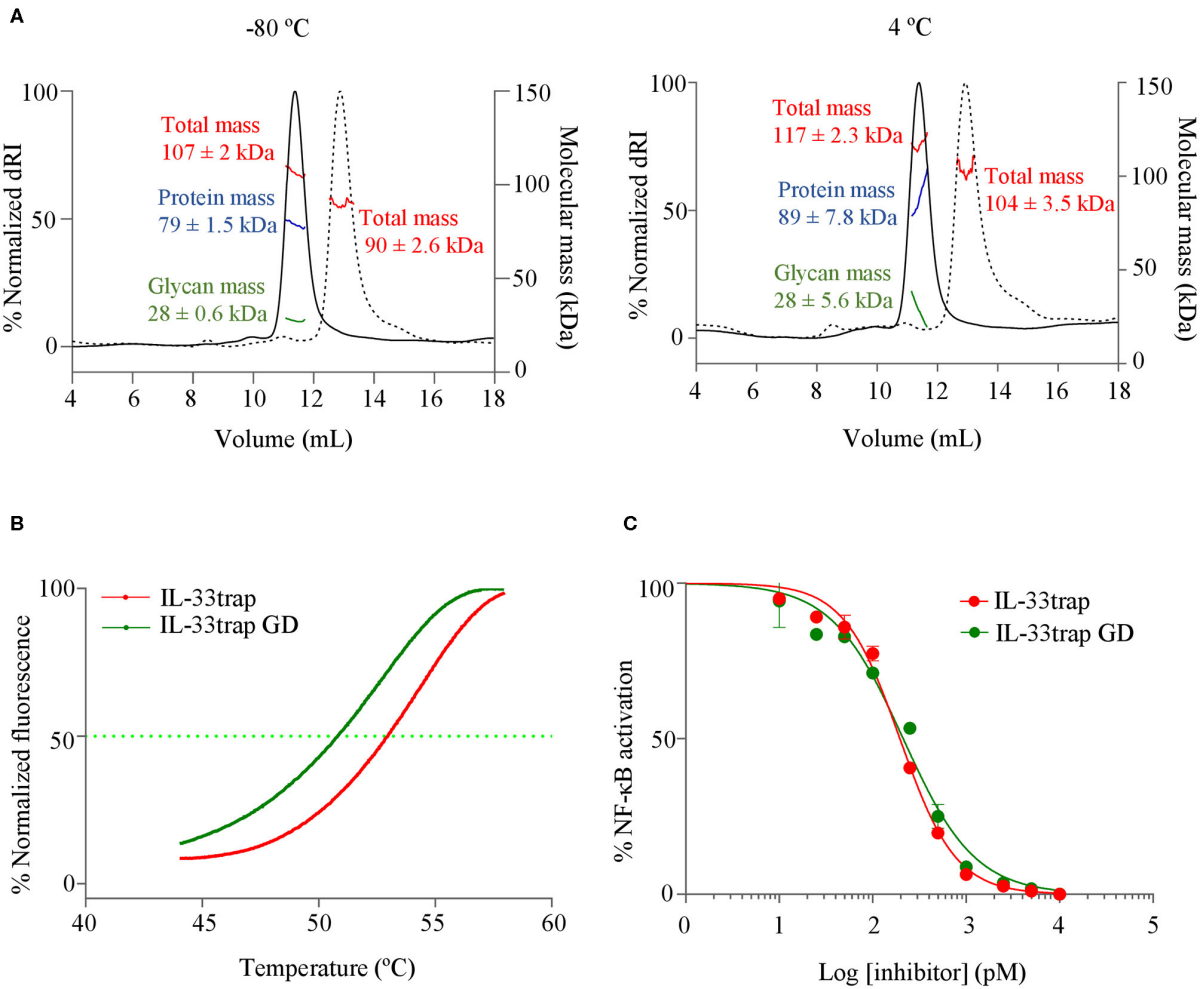
Glycosylated and under-glycosylated murine IL-33trap do not form aggregates and are biophysically stable during liquid storage and at physiological temperature. **(A)** SEC-MALLS analysis of murine 20xGGS linker-containing IL-33trap expressed in HEK293T or HEK293 GlycoDelete cells (referred as IL-33trapGD) and frozen-stored vs. liquid-stored at 4°C for a time period of 10 days. SEC elution profiles of IL-33trap (full line) and IL-33trapGD (dashed line) are plotted as the normalized differential refractive index (dRI, left vertical axis) as a function of the elution volume (mL). Molecular weight of IL-33trap and IL-33trapGD as determined by MALLS (kDa, right vertical axis) is dissected by protein conjugate analysis in total (red), protein (blue) and sugar (green) mass and is reported as the number-average molar mass across the elution peak ± SD. Only the total mass (red) of IL-33trapGD is reported as protein conjugate analysis did not yield significant glycan mass. *n* = 1 sample injected. SEC-MALLS data was analyzed using ASTRA6 software (Wyatt) and graphs were plotted using GraphPad Prism software. **(B)** Melting curves of murine IL-33trap and IL-33trapGD plotted as normalized fluorescence intensity in function of increasing temperature, determined by ThermoFluor® assay. Values of melting temperature (T_m_) and onset temperature (T_o_) were calculated using GraphPad Prism software. **(C)** Comparison of the effect of IL-33trap and IL-33trapGD on IL-33 induced NF-κB activation. HEK293T cells were treated with recombinant murine IL-33 that was pre-incubated with murine IL-33trap or IL-33trapGD and assayed for NF-κB activity as described in Material and Methods. Values represent means ± SE of technical triplicates. Results are representative of at least two independent experiments.

The biophysical behavior of therapeutic proteins is very important for the correct development and optimisation of biologics, since it may impact many aspects of drug function, stability and activity. Excessive aggregates and fragmentation, as well as denaturation or oxidation are indicative of an unstable product, unsuitable for *in vivo* use. For this reason, we sought to characterize the molecular and thermal stability of the murine 20xGGS-linker containing but Fc-less IL-33trap for which we previously showed an inhibitory effect upon local delivery in a mouse asthma model (15). First, we examined whether IL-33trap is prone to form aggregates during storage. IL-33trap was stored frozen in PBS at −80°C or liquid-stored at 4°C for a time period of 10 days. To determine the presence of potential aggregates, we used size-exclusion chromatography (SEC) coupled with ultraviolet (UV), multi-angle light scattering laser (MALLS) and refractive index (RI) detectors. Protein elution fractions were identified as eluting species with a peak in both UV absorbance and differential RI (dRI) intensity. IL-33trap, either frozen-stored at −80°C or liquid-stored at 4°C for 10 days, was found to be highly homogeneous, adopting monodisperse assemblies, with a protein molecular mass comparable to the theoretical (Figure 2A).

We next assessed the thermostability of IL-33trap using ThermoFluor® assay (19), and melting temperature (T_m_) was calculated as the V50 value of a Boltzmann sigmoidal curve fitted to the melting curve (19, 22). The analysis showed a T_m_ and a T_o_ of 55 and 46.5°C, respectively which is well above the physiological temperature of 37°C (Figure 2B). Together, our data indicate that IL-33trap is a stable molecule at physiological temperature and does not form aggregates when liquid-stored at 4°C for up to 10 days.

### Glycosylation Does Not Affect IL-33trap Bioactivity

We previously showed that mouse IL-33trap expressed in HEK293T cells is glycosylated (15). To further estimate the degree of glycosylation, we calculated the total protein and glycan molecular weight on IL-33trap using SEC-MALLS and ASTRA6 software (Wyatt), showing that IL-33trap is heavily glycosylated (25–35% of total mass) (Figure 2A). Using NetNglyc 1.0 Server and NetOglyc 4.0 Server prediction (23), 15 potential N-glycosylation and 1–4 potential O-glycosylation sites were found. Although not all of these sites are likely to be occupied, the high number of glycosylation sites leads to significant product heterogeneity, which is further complicated by the inherent difference in glycan chain length and complexity in eukaryotic expression systems. This is a major challenge for efficient purification at high yield, to set sound specifications for product release and hence to assure batch reproducibility. It is also likely to attribute significant heterogeneity in pharmacokinetic behavior of the molecule, due to differential lectin-mediated blood clearance of different glycoforms. Therefore, we decided to reduce the glycosylation complexity and heterogeneity of mouse IL-33trap using HEK293 GlycoDelete technology (18), which reduces glycosylation to short single-branch oligosaccharides that are partially sialylated. Indeed, IL-33trap produced in HEK293 GlycoDelete is notably less glycosylated (6% of the total mass) compared to the original IL-33trap molecule produced in HEK293 FreeStyle cells (25–35% of the total mass) (Figure 2A). We then compared the thermostability and antagonistic activity of the glycosylated and under-glycosylated IL-33trap variants. Reduced glycosylation had only a mild effect on the melting temperature (Figure 2B), and did not affect the ability of IL-33trap to inhibit IL-33-induced NF-κB activation (Figure 2C).

### IL-33trap Specifically Targets Active IL-33 Splice Variants

**Figure 3:**
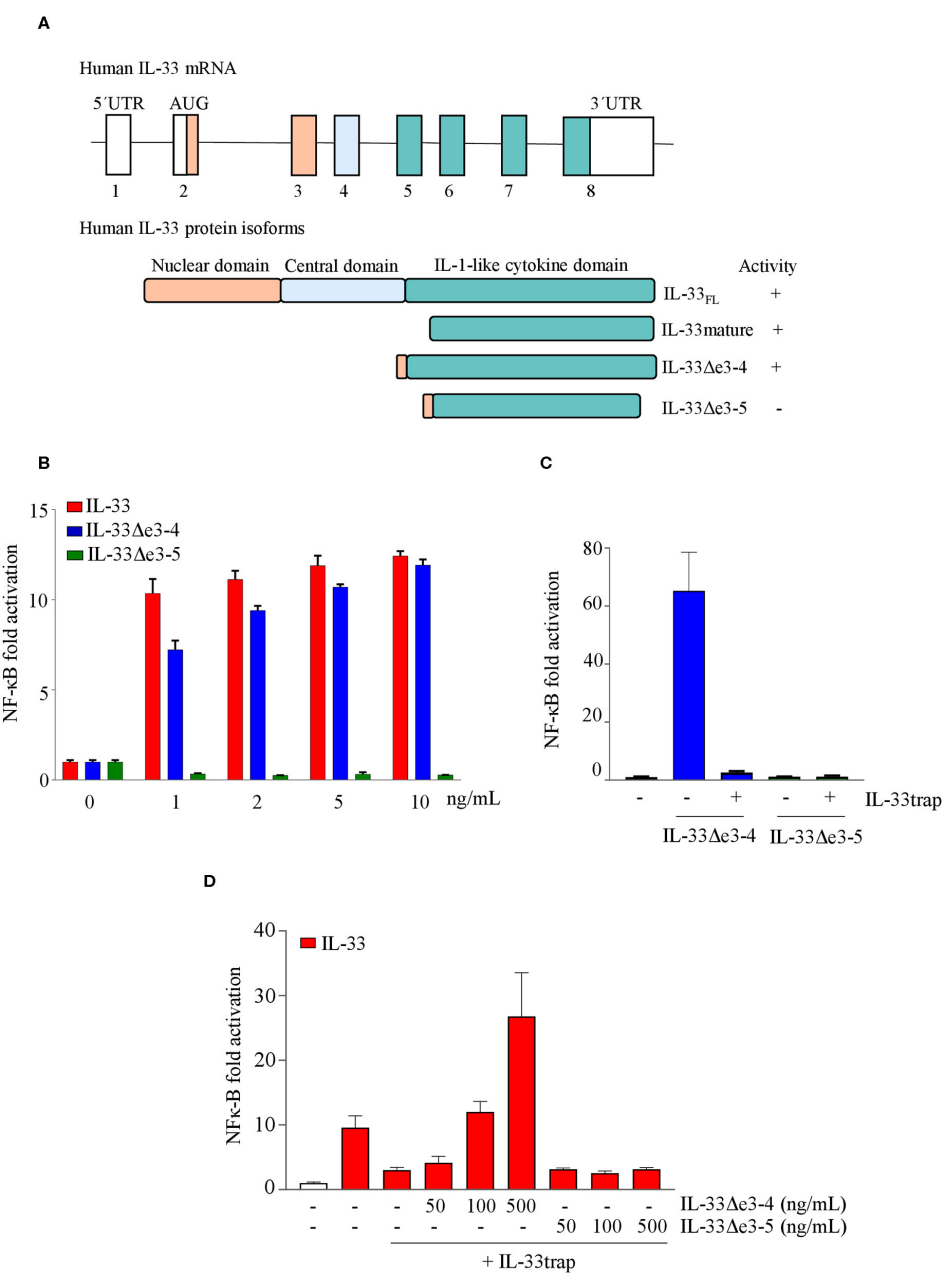
Human IL-33trap specifically targets active IL-33 splice variants **(A)** Schematic representation of human IL-33 splice variants and encoded protein isoforms. **(B)** NF-κB activation induced by different IL-33 splice variants. **(C)** Effect of IL-33trap on NF-κB activation induced by different IL-33 splice variants. **(D)** Effect of the presence of IL-33 splice variants on the ability of IL-33trap to inhibit IL-33 induced NF-κB activation. NF-κB activity was measured in HEK293T cells as described in Material and Methods. Values represent means ± SE of technical triplicates. Results are representative of at least two independent experiments.

Human IL-33 mRNA can be alternatively spliced in several smaller IL-33 splice variants lacking exon 3, 4, 5 or a combination thereof, which are expressed in different cell types in different proportions (24). In contrast to deletion of exons 3 and 4, absence of exon 5 results in loss of IL-33 activity (25), which is consistent with the fact that exon 5 encodes amino acid residues that are critical for ST2 binding (26). Importantly, binding of inactive IL-33 isoforms to IL-33-neutralizing biologics would serve as a natural sink and decrease drug availability. However, because IL-33trap is a receptor-based biologic, it is expected to exclusively interact with bioactive receptor-binding IL-33 isoforms and to be insensitive to the presence of inactive IL-33 splice variants. To further test this hypothesis, we generated IL-33 splice variants lacking exons 3 and 4 (IL-33Δe3-4) or exons 3, 4 and 5 (IL-33Δe3-5) (Figure 3A), and analyzed their activity in an IL-33 bioassay. As shown previously, IL-33Δe3-4 efficiently induced NF-κB activation, while IL-33Δe3-5 was inactive (Figure 3B). Furthermore, NF-κB activation induced by IL-33Δe3-4 was efficiently inhibited by IL-33trap (Figure 3C). We next performed a competition assay, where we pre-incubated mature IL-33 with IL-33trap in the presence of increasing concentrations of either IL-33Δe3-4 or IL-33Δe3-5 splice variants. Consistent with its receptor-binding capacity, addition of IL-33Δe3-4 reduced the ability of IL-33trap to inhibit IL-33 signaling, while addition of IL-33Δe3-5 had no effect (Figure 3D). These results illustrate that inactive IL-33 splice variants will not act as a sink for IL-33trap, which might offer a significant advantage compared to certain monoclonal antibodies that, depending on the recognized epitope, might not always distinguish between active and inactive IL-33 isoforms.

### Generation and Validation of a Human IL-4/13 Trap as a Dual Cytokine Antagonist

**Figure 4:**
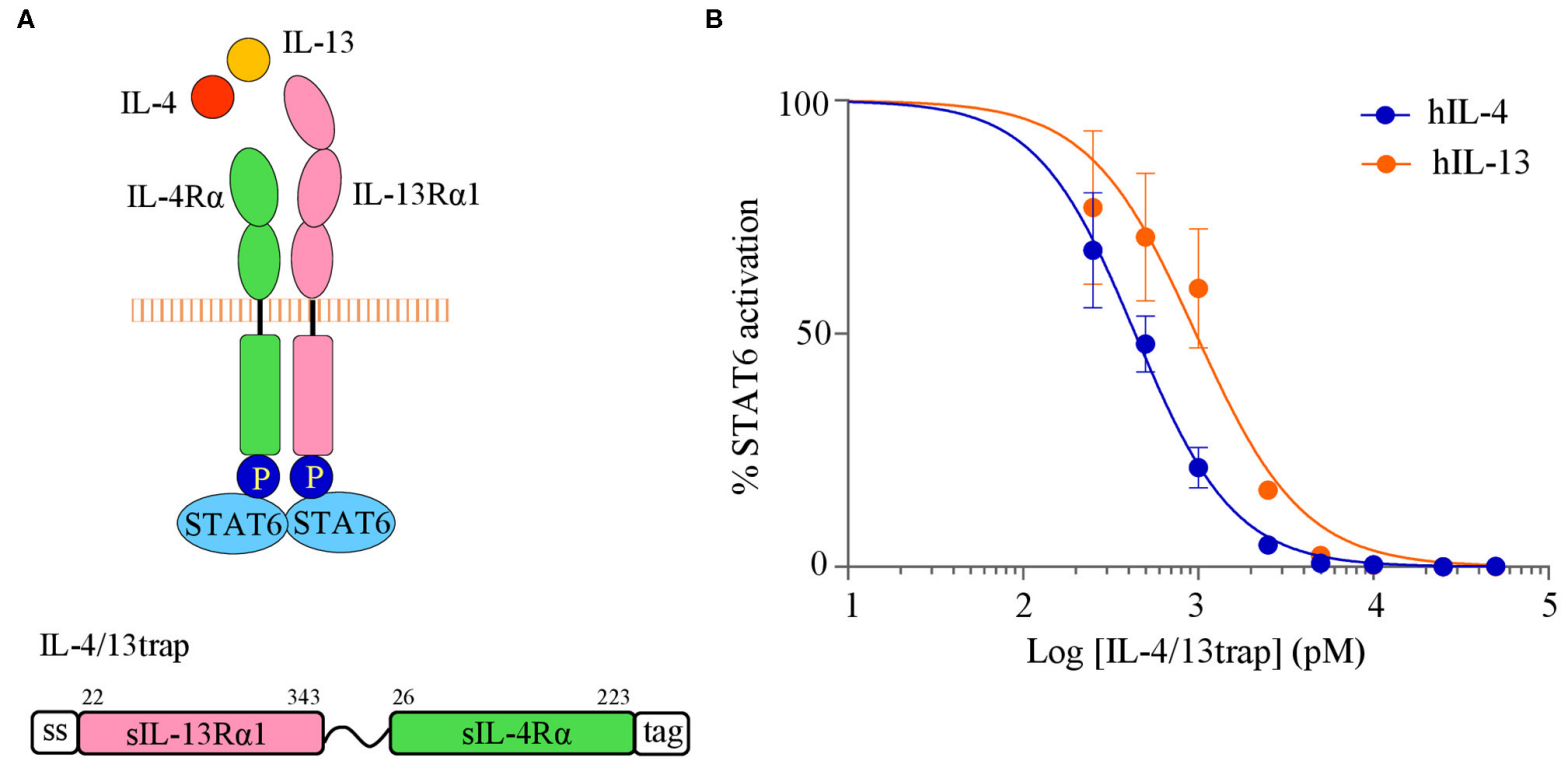
Human IL-4/13trap inhibits IL-4 and IL-13 activity. **(A)** Schematic representation of the type II receptor complex for IL-4 and IL-13 (also receptor phosphorylation and recruitment of STAT6 is shown), and the corresponding human IL-4/13trap construct (numbers indicate the amino acid boundaries of the receptor ectodomains). **(B)** Effect of human IL-4/13trap on IL-4- and IL-13-induced STAT6 activation. HEK293T cells were treated with recombinant human IL-4 or IL-13 that was pre-incubated with human IL-4/13trap and assayed for STAT6 activity as described in Material and Methods. Values represent means ± SE of technical triplicates. Results are representative of at least two independent experiments.

As for the IL-33trap, a similar approach using single-chain soluble receptor fusion proteins can be used to target other cytokines that signal through a heterodimeric receptor complex. In this regard, we have previously reported the design and validation of TSLP-trap, a fusion protein which consists of the extracellular domains of TSLPR and IL-7Rα fused via a 20xGGS flexible linker (27). Together with IL-33 and IL-25, the epithelium-derived cytokine TSLP is considered a central orchestrator of Th2 responses in atopic disorders, and therefore a promising therapeutic target. Similarly, IL-4 and IL-13 are in the spotlight as interesting dual therapeutic targets in type 2-driven inflammatory disease (28). Importantly, these two cytokines both bind to the type II receptor consisting of IL-4 receptor alpha (IL-4Rα) and IL-13Rα1 (29) (Figure 4A), offering a possibility to simultaneously target two cytokines with one inhibitor. Using a similar design as IL-33trap (15), we generated and validated a new human IL-4/13trap, consisting of the extracellular domain of human IL-13Rα1 fused to the extracellular domain of human IL-4Rα via a flexible 20xGGS linker. The expression construct also contains the human IL-13Rα1 signal sequence at the N-terminus, which allows protein secretion, and a myc/His tag at the C-terminus to facilitate protein purification and detection (Figure 4A). Human IL-4/13trap was produced using HEK293 FreeStyle cells to ensure proper folding, and purified from conditioned media using immobilized metal affinity chromatography and size exclusion chromatography, as described in materials and methods.

To test whether IL-4/13trap displays antagonistic properties, we investigated its ability to inhibit both IL-4 and IL-13 signaling in a cell-based assay. To this end, HEK293T cells were made responsive to either IL-4 or IL-13 by transfection with human IL-4Rα and IL-13Rα1, and downstream STAT6 activation was followed via STAT6-dependent luciferase reporter gene expression. IL-4 as well as IL-13 treatment resulted in robust STAT6 activation (Figure 4B). Importantly, pre-incubation of either IL-4 or IL-13 with equimolar concentration of IL-4/13trap strongly reduced IL-4- and IL-13-induced STAT6-dependent luciferase activity (IC_50_ of 465 pM and 434 pM, respectively) (Figure 4B). These data convincingly demonstrate that IL-4/13trap behaves as a strong IL-4 and IL-13 antagonist and illustrate the feasibility to develop recombinant single-chain soluble receptor fusion proteins as novel biologics for the inhibition of a wide range of cytokines or other protein ligands that signal via a heterodimeric receptor complex.

## Discussion

Because of the important role of cytokines in human autoimmune and inflammatory diseases, many biologics targeting cytokines and their receptors have been developed over the past years. Several soluble receptor-based biologics, such as the TNF antagonist Etanercept, the IL-1 antagonist Rilonacept, and the VEGF antagonist Aflibercept, are already actively used in the clinic as alternatives for monoclonal antibodies. In all cases, soluble receptors were engineered to encode an IgG Fc region to increase half-life and to permit dimerisation and high affinity ligand binding. A similar approach has been described for IL-4 and IL-6 neutralizing trap molecules (30), which have not yet entered the clinic. The use of Fc fusion to enable dimerisation doubles the size of the inhibitor (> 250 kDa in the case of Rilonacept, glycosylation not included), limiting its tissue permeability, which may be even more critical in certain conditions such as asthma where mucus imposes an additional barrier. Moreover, larger proteins typically result in lower expression levels while higher dosing is needed compared to smaller proteins (~90 kDa in the case of IL-33trap) to achieve the same molar concentration. The presence of an Fc portion could also lead to side effects due to nonspecific binding to Fc receptors or Fc-associated effector functions, although Fc engineering can also overcome such problems. However, it remained unclear whether Fc-fusion-driven bivalency also contributes to the cytokine neutralizing activity of soluble receptor fusion proteins like the IL-33trap. We have demonstrated here that the Fc moiety strongly enhances the inhibitory capacity of a linker-less IL-33trap molecule, indicating a role for Fc-mediated dimerisation in the formation of a fully functional soluble receptor complex. The need for Fc-mediated dimerisation could however be completely replaced by the introduction of a flexible linker (12xGGS) between both receptor subunits, which allowed the formation of a single-chain fusion protein with an optimal conformation for high affinity ligand binding. The use of a flexible linker in the design of IL-33trap thus offers a significantly different candidate biotherapeutic as an alternative to Fc fusion. The use of Fc-less fusion biologics could be especially relevant for maximizing exposure of the therapeutic in certain tissues like the eye and lung upon local delivery, where FcRn-mediated transcytosis of Fc-fusion proteins across epithelial and endothelial cells would otherwise mediate transport into systemic circulation.

The manufacturability of therapeutic proteins entails major challenges to ensure drug efficacy and safety. Therefore, appropriate molecular characterization and optimisation of these biologics is crucial to ensure protein stability, homogeneity, low immunogenicity as well as optimal pharmacokinetic properties. Therapeutic proteins are often susceptible to thermal stress, which can cause drastic conformational changes resulting in reduced drug efficacy and stability. Furthermore, protein unfolding *in vivo* may favor intermolecular protein interactions and subsequent aggregates formation, which in turn could trigger immune responses inducing formation of anti-drug antibodies (31, 32). SEC-MALLS analysis indicated that IL-33trap does neither form aggregates nor undergo denaturation above physiological temperatures. Moreover, IL-33trap stability was not affected during liquid storage, which may be of interest for clinical practices as it might facilitate patient self-administration. The quality of protein-based biologics may also be determined by protein glycosylation. Inherent differences in glycan chain length and complexity in eukaryotic expression systems can result in highly heterogeneous and complex glycosylation pattern (33). This is a major challenge to ensure efficient purification at high yield and batch reproducibility. Our data show that reducing glycosylation complexity and heterogeneity of IL-33trap using HEK293 GlycoDelete technology does not affect its bioactivity.

The complexity of cytokine networks is drastically increased by the generation of multiple cytokine and cytokine receptor isoforms due to alternative splicing, differential promotor usage, or posttranslational modifications such as proteolytic cleavage and degradation (34, 35). This increases the risk that certain active isoforms or variants may escape recognition by epitope-specific monoclonal antibodies, leading to drug resistance. Unfortunately, the specific epitopes that are recognized by monoclonal antibodies that are in clinical development have not been reported in literature. Alternatively, inactive isoforms that do bind monoclonal antibodies may act as a sink, again reducing that efficacy of the biologic. Also in the case of IL-33, alternative splicing (24) and proteolytic cleavage (34) have been described, creating tens of active and inactive IL-33 isoforms. Possibly, some of these variants may escape or interfere with currently used IL-33 neutralizing monoclonal antibodies, which may lead to poor responses in preclinical or clinical studies. Importantly, as the here described soluble receptor-based cytokine traps fully mimic cytokine-binding by endogenous cell surface receptors, they are expected to neutralize all biologically active cytokine isoforms and be insensitive to the presence of inactive isoforms, as also documented in the present study in the case of specific IL-33 splice variants.

Although targeting of specific cytokines with receptor-based fusion proteins and monoclonal antibodies have demonstrated beneficial clinical outcomes in patients suffering from a wide variety of inflammatory disorders (1), functional redundancy of cytokines as well as the development of anti-drug antibodies often limits the success of specific cytokine therapies. Thus, combinatorial treatment approaches simultaneously targeting several cytokines may improve clinical outcomes, which has also been documented for IL-33 and TSLP in preclinical studies (36). Therefore, combination of IL-33trap with TSLPtrap, which we have previously described as a 20–30 fold more potent TSLP inhibitor *in vitro* than the anti-TSLP antibody Tezepelumab (27), might be an interesting therapeutic approach. Dual targeting of IL-4 and IL-13 with Dupilumab, a monoclonal antibody targeting IL-4Rα, has recently entered the clinic for certain allergic diseases (37, 38). Likewise, a novel bispecific llama-based antibody simultaneously targeting IL-4Rα and IL-5, providing a triple blockade of IL-4, IL-13 and IL-5 signaling, has been developed (39). Of interest, simultaneous inhibition of IL-13 and IL-33 signaling was shown to inhibit allergic airway inflammation in mice more effectively than inhibition of either cytokine alone (40). In the present study we have shown that our design of single-chain soluble receptor fusion proteins for the development of cytokine traps is not only applicable to IL-33 and TSLP, but also to IL-4 and IL-13. It will therefore be of interest to further test the effect of the here described IL-4/13trap in preclinical mouse models. Also, the generation of multi-specific cytokine trap therapeutics, by fusing different cytokine trap proteins, might be a path worth to consider. So far, only murine IL-33trap has been tested *in vivo* in a mouse asthma model (15). It will be interesting to also develop clinically relevant *in vivo* models where the human IL-33 trap can be tested. In preliminary experiments we have observed that repetitive intratracheal administration of human IL-33 in mice induces lung eosinophilia, which can be prevented by treatment of mice with human IL-33trap. Use of humanized mice reconstituted with human immune cells (41), which are then intratracheally injected with human IL-33, might also be an option. Ideally, one might use humanized mice in which the murine *IL-33* gene has been replaced by the human *IL-33* gene, which would allow to test the effect of human IL-33trap in an allergic disease mouse model that relies on human IL-33 production from epithelial or endothelial cells. However, it remains to be seen if such a human IL-33 transgene will be regulated similarly to the mouse gene in a murine context. Similar approaches might be applicable for preclinical studies using IL-4/13trap.

In conclusion, following the quest for additional novel anti-cytokine biologics, our data illustrate the potential of recombinant single-chain soluble receptor fusion proteins as novel anti-cytokine biologics. Cytokine traps targeting IL-33, TSLP, IL-4 and IL-13 are novel tools that nicely complement the use of monoclonal antibodies for the treatment of allergic diseases. Moreover, the translational impact of such therapeutics can be expected to be much broader than allergic diseases.

## Data Availability Statement

The datasets generated for this study are available on request to the corresponding author.

## Author Contributions

AH, HB, and IA designed the experiments. AH, HB, DV, and IA performed experiments. NC provided the HEK293 GlycoDelete cells and associated protein production protocols. SS and KV helped with the biophysical characterization of the IL-33trap. AH, IA, and RB wrote the manuscript. RB and IA supervised the work. All authors contributed to the scientific discussion.

## Conflict of Interest

RB, HB, and SS are inventors on patents related to IL-33trap and TSLPtrap. The remaining authors declare that the research was conducted in the absence of any commercial or financial relationships that could be construed as a potential conflict of interest.
